# Fatigue Performance Prediction of RC Beams Based on Optimized Machine Learning Technology

**DOI:** 10.3390/ma15186349

**Published:** 2022-09-13

**Authors:** Li Song, Lian Wang, Hongshuo Sun, Chenxing Cui, Zhiwu Yu

**Affiliations:** 1 School of Civil Engineering, Central South University, Changsha 410082, China; 2 National Engineering Research Center of High-Speed Railway Construction Technology, Changsha 410075, China

**Keywords:** deep belief network, particle swarm optimization, reinforced concrete beam, fatigue performance, structural damage, BP neural network

## Abstract

The development of fatigue damage in reinforced concrete (RC) beams is affected by various factors such as repetitive loads and material properties, and there exists a complex nonlinear mapping relationship between their fatigue performance and each factor. To this end, a fatigue performance prediction model for RC beams was proposed based on the deep belief network (DBN) optimized by particle swarm optimization (PSO). The original database of fatigue loading tests was established by conducting fatigue loading tests on RC beams. The mid-span deflection, reinforcement strain, and concrete strain during fatigue loading of RC beams were predicted and evaluated. The fatigue performance prediction results of the RC beam based on the PSO-DBN model were compared with those of the single DBN model and the BP model. The models were evaluated using the *R*^2^ coefficient, mean absolute percentage error, mean absolute error, and root mean square error. The results showed that the fatigue performance prediction model of RC beams based on PSO-DBN is more accurate and efficient.

## 1. Introduction

As important components of the global land transportation network, bridges, the most common of which are reinforced concrete (RC), play a pivotal role in improving people’s livelihoods and promoting regional economic development [1]. In the context of increasing traffic volume, RC beams are subjected to high-frequency vehicle loads for a long time. The service performance and durability performance of many bridges have deteriorated at an accelerated rate, and the fatigue life has been further reduced, which seriously affects the operational safety of RC bridge structures [2,3]. From a macroscopic perspective, the fatigue damage of RC beams is caused by the gradual accumulation of structural material damage and the gradual deterioration of properties, and the fatigue performance of structural materials determines the fatigue performance of the structure itself [4]. The adoption of scientific and reliable methods to effectively evaluate the fatigue performance of existing RC beams has become an urgent engineering problem to be solved.

At present, the fatigue performance of RC beams has been studied mainly through indoor fatigue loading tests [5,6,7]. Wang and Li [8] investigated the effect of material randomness on structural fatigue performance by conducting fatigue loading tests on RC beams. Yang et al. [9] studied the fatigue performance of RC beams after water freeze-thaw and salt freeze-thaw cycles by conducting four-point bending fatigue loading tests. There are problems such as high intensity, long cycle time, and difficulty in obtaining fast and accurate test results in a short period of conducting a large number of tests. With the continuous development of numerical simulation technology, more and more researchers have started to use numerical simulation methods to study the fatigue performance of RC beams [10,11,12]. Jin et al. [13] simulated the bond-slip behavior of longitudinal reinforcement by establishing a three-dimensional mesoscale numerical model and investigated the mechanical properties of carbon fiber reinforced polymer (CFRP)-reinforced RC beams with impact damage. He et al. [14] studied the fatigue performance of ordinary RC beams and reinforced beams by simulating impact tests, and the dynamic properties of RC beams before and after CFRP strengthening under different impact conditions were investigated. However, the fatigue life of RC beams can be predicted well by using a single fatigue analysis software, but the mechanical properties of RC beams after a certain number of cyclic loading cannot be accurately evaluated, and it is difficult to realize the whole process of fatigue analysis. Dobromil P. et al. [15] used the finite element method to simulate the fatigue damage process of concrete structures, but usually, the accurate mechanical performance analysis needs to be realized by writing special programs or secondary development of software, and the versatility is not strong. The number of fatigue loading is usually in the millions, and it is a huge workload to measure the mechanical properties of RC beams under different loading times in a turn. Therefore, it is important to combine the appropriate amount of indoor fatigue loading tests with efficient scientific calculation algorithms to effectively judge the mechanical properties of RC beams under different damage conditions after cyclic loading for engineering structural evaluation and fatigue durability research.

In recent years, with the continuous development of artificial intelligence technology, many scholars have applied artificial neural networks in engineering structure prediction or structural material research [16,17,18,19,20,21,22] with rich results. Liu et al. [23] established a BP neural network prediction model for blast safe vibration velocity of newly cast concrete structures based on BP neural network theory and selected key influencing factors such as Poisson’s ratio, and predicted the blast safe vibration velocity of concrete at different ages under two different conditions. Asteris et al. [24] used the artificial neural network method to predict the ultimate shear strength of RC beams and compared the predicted values with the experimental values as well as the values calculated by existing formulas in the code provisions, which proved the reliability and validity of the predictive performance of artificial neural networks. Cong et al. [25] used neural networks to estimate the effluent water quality at frequent changes in conditions to improve the accuracy of water quality evaluation in the wastewater treatment process. Sahoo and Mahapatra [26] used an artificial neural network (ANN) model to predict the compressive strength of concrete at different water curing days, sulfate exposure time, and fly ash substitution levels. Onyari and Ikotun [27] used an artificial neural network to predict the compressive and flexural strength of modified zeolite additive mortar, and the results showed that the compressive and flexural strength of modified zeolite mortar could be very short using a neural network model and the prediction results are more accurate.

The deep learning method, as the frontier in the field of machine learning, is one of the commonly used methods in the field of artificial intelligence. It improves prediction accuracy by building models with multiple layers and mining the features implicit in the learned data. Zhang et al. [28] proposed a general method for component life prediction under creep, fatigue, and creep-fatigue conditions based on deep learning. It has better prediction accuracy and generalization ability than traditional machine learning models. Yang et al. [29] proposed a multi-axis fatigue life prediction model based on deep learning and analyzed six sets of existing fatigue data from different materials, respectively. Hinton et al. [30] proposed a deep learning method called deep belief network (DBN), which has attracted a lot of attention from the academic community. DBN is a stack of multiple restricted Boltzmann machines (RBMs), which can map complex nonlinear relationships and have a better-unsupervised feature learning capability, and can maintain strong stability when dealing with complex data prediction [31]. Currently, DBN has been successfully applied to problems such as identification or evaluation in engineering. Wang et al. [32] based a regional landslide sensitivity zoning model on a deep belief network model with example analysis and finally compared the evaluation results with logistic regression (LR) and artificial neural network (BPNN) model evaluation results by RO curve features. Chen et al. [33] evaluated the landslide susceptibility of a region using the DBN model, generated a landslide susceptibility zoning map of the region, and compared it with shallow neural networks and traditional logistic regression methods. Xu et al. [34] identified RC beam damage based on acoustic emission and DBN.

Although the deep belief network is widely used, it has problems in that the number of hidden layer nodes is not easily determined, and the parameters, such as learning rate and the number of iterations, are mainly determined by manual experience during the pre-training process, and improperly set parameter values can have an impact on the model prediction performance. Therefore, it is necessary to optimize the DBN model further to further improve the accuracy of prediction. In this paper, a particle swarm optimization algorithm is used to search for the optimal number of hidden layer nodes. At present, regarding the intelligent search algorithms, the common ones are the simulated annealing algorithm [35], genetic algorithm [36,37], and particle swarm optimization algorithm. Among them, the effect of global optimization and computational efficiency of simulated annealing algorithm is greatly affected by parameters, genetic algorithm is difficult to converge effectively in a limited time, and particle swarm optimization algorithm is a population-based evolutionary algorithm with simple and easy-to-understand principle, fast convergence speed and global optimal solution.

In this paper, the fatigue performance of RC specimen beams was analyzed by conducting a four-point bending fatigue loading test under constant amplitude load, and the original database was established. A deep belief network (DBN) model for fatigue performance prediction of RC beams was established for the first time by using deep learning with massively parallel processing and self-learning characteristics. After training the RBM layer by layer and extracting feature information from complex data, the DBN model parameters were adaptively adjusted using a particle swarm optimization algorithm. The results of the fatigue performance prediction of RC beams based on the PSO-DBN model were compared with those of the single DBN model and the BP model. The comparative analysis of the simulation model predictions verified the feasibility and accuracy of the fatigue performance prediction of RC beams based on the PSO-DBN model and provided new ideas for engineering structure evaluation and fatigue durability research.

## 2. Experimental Program

### 2.1. Specimens

**Figure 1:**
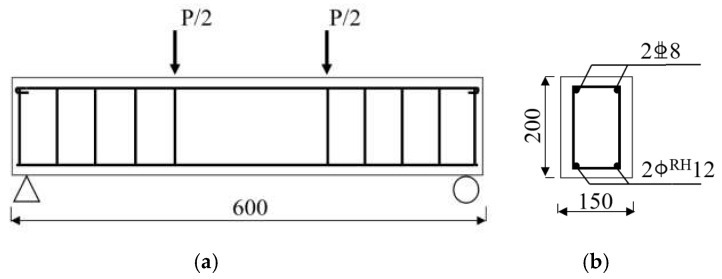
Details of test specimens (unit: mm): (**a**) Geometry; (**b**) Midspan section.

**Figure 2:**
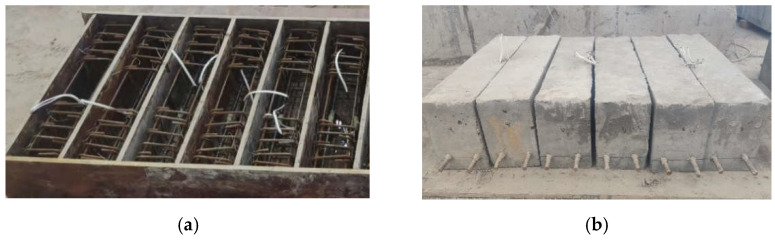
Specimen beams: (**a**) Before concreting; (**b**) After concreting.

Six RC specimen beams were designed for this test, and the design strength grade of concrete for the specimen beams was divided into three types, C35, C40, and C60 (150 mm cubic compressive strengths are 35 MPa, 40 MPa, and 60 MPa at the age of 28 days), two of each type, one of which was used for a static loading test and the remaining one for a fatigue loading test. The cross-sectional dimensions of the specimen beam were 150 mm × 200 mm, with a total length of 600 mm, and the tensile reinforcement was CRB600H (a new type of cold-rolled ribbed steel bar developed in China in recent years) with high strength and high ductility. The specific arrangement of the reinforcement is shown in Figure 1. Figure 2 shows the specimen beams before and after concreting.

### 2.2. Material Properties

**Figure 3:**
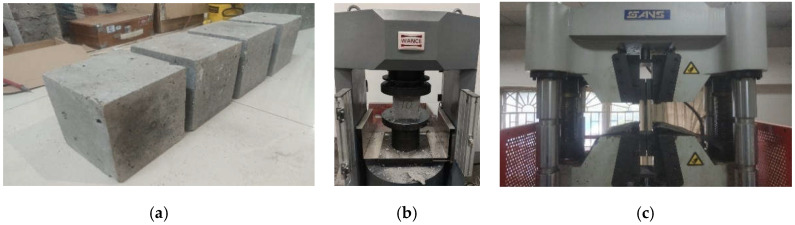
Material performance testing: (**a**,**b**) physical and mechanical properties test of concrete; and (**c**) material mechanical properties test of reinforcement.

The mechanical properties of materials were tested, as shown in Figure 3. According to the Standard for Test Methods of Physical and Mechanical Properties of Concrete (GB/T 50081-2019) [38], three standard cubic specimens were reserved for each of the three types of concrete with different design strengths during the casting of the specimen beams, and after the specimens were cured for 28 days, the average values of their cubic compressive strengths were tested to be 36.9 MPa, 55 MPa, and 63.7 MPa, respectively. The concrete mix ratios are shown in Table 1. According to the Metal Axial Tensile Test Method (GB/T 228.1-2010) [39], three pieces of each type of reinforcement were reserved for material mechanical property tests. The average yield strength and ultimate tensile strength of CRB600H reinforcements with a nominal diameter of 12 mm were 619 MPa and 671 MPa, respectively, and the average yield strength and ultimate tensile strength of HRB400 reinforcements with a nominal diameter of 8 mm were 467 MPa and 560 MPa, respectively.

### 2.3. Test Loading Device and Loading System

**Figure 4:**
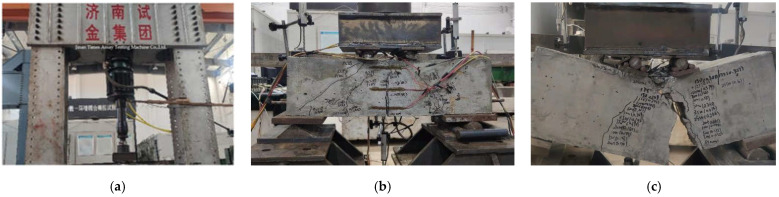
Test loading process: (**a**) test loading device; (**b**) specimen beams static loading test; (**c**) specimen beams fatigue loading test.

After finishing the maintenance of the specimen beams, the PMS-500 hydraulic pulsation testing machine produced by China Jinan Times Assay Testing Machine Co., Ltd. was used for test loading, and the loading device is shown in Figure 4a. The test included a static loading test and fatigue loading test, using vertical force control loading and four-point bending cycle constant amplitude loading, respectively. The purpose of the static loading test was to determine the static ultimate bearing capacity of the RC specimen beams and then determine the upper limit value of the fatigue load of the RC specimen beams, and the static test loading is shown in Figure 4b. Before the official start of the static loading test, it was preloaded to 10 kN to promote sufficient contact between the couplings. After checking that the channels of each measurement point were normal, the loading was carried out in increments of 10 kN per level of load, and the load increment per level was appropriately reduced when the specimen beams were close to damage. The indicators, such as mid-span deflection, the strain of tensile reinforcement, and concrete strain in the compression zone at the top of the beam, were measured during the test loading. With the increasing load, the tensile reinforcement at the bottom of the RC-1, RC-3, and RC-5 specimen beams yielded, and the concrete at the top of the beams was crushed. Finally, the static ultimate bearing capacities of the three specimen beams were 205 kN, 232 kN, and 243 kN, respectively.

The fatigue test used four-point bending equal amplitude load, and the fatigue test loading is shown in Figure 4c. According to the results of the static loading test, the upper limit of fatigue load for RC-2, RC-4, and RC-6 specimen beams was taken as 40 kN, 70 kN, and 90 kN, and the lower limit of fatigue load was taken as 10 kN, and the fatigue lives of the three specimen beams were 3.59 million, 2.76 million, and 3.28 million times, respectively. The test loading scheme is shown in Table 2.

### 2.4. Measurement Point Arrangement and Data Acquisition

Before the RC specimen beams were cast, reinforcement strain gauges were arranged on the tensile reinforcement in the middle of the span of the beam to measure the strain of the tensile reinforcement in the specimen beams. Before the test, two concrete strain gauges were arranged at the top of the span of the specimen beams to measure the strain in the concrete of the specimen beams. Displacement sensors were arranged at the bottom of the span of the specimen beams and the top surface of the beams above the support to record the vertical displacement and the support settlement, respectively. During the loading process of the fatigue test, the DH3820 high-speed static strain testing and analysis system produced by China Jiangsu Donghua Testing Technology Co., Ltd. was used to automatically collect the deflection, strain on the tensile reinforcement, and strain on the concrete top of the beams during the test.

### 2.5. Test Results and Analysis

**Figure 5:**
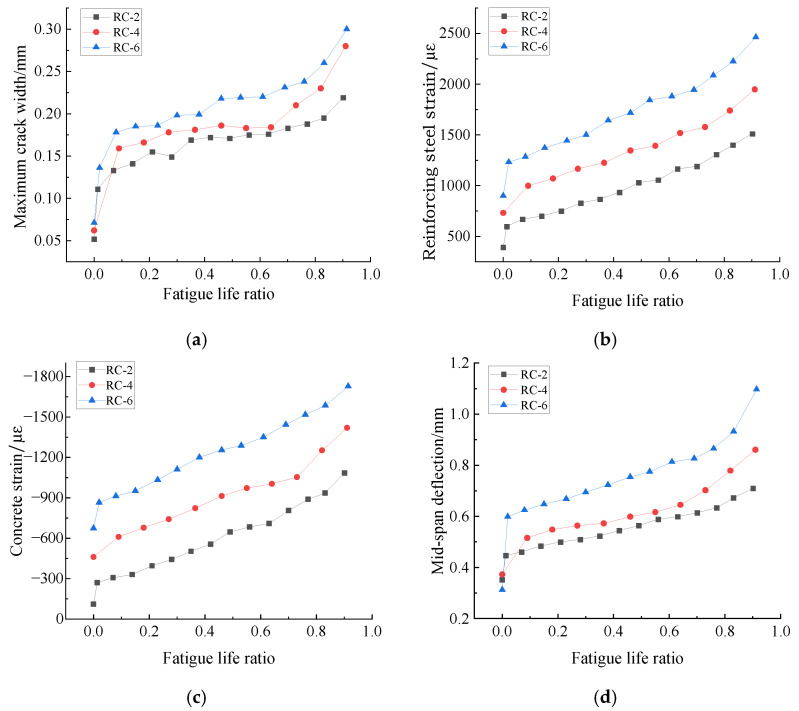
Fatigue performance of RC specimen beams under different numbers of cyclic loading: (**a**) maximum crack width; (**b**) tensile reinforcement strain; (**c**) concrete strain; (**d**) mid-span deflection.

Figure 5 shows the fatigue performance of RC-2, RC-4, and RC-6 specimen beams under different load cycles. The relationship curves between the maximum crack width and fatigue life ratio n/N of the specimen beams are shown in Figure 5a. From Figure 5a, it can be seen that the crack development process of the specimen beams shows a "three-stage" pattern. The first stage is the crack derivation stage, in which the crack width is about 0–0.175 mm, the second stage is the stable crack development stage, in which the crack width is about 0.175–0.25 mm, and the third stage is the fatigue damage stage, in which the crack width expands sharply with the increase of the number of cycles, and finally leads to the fatigue damage of the specimen beams.

The curves of the bottom tensile reinforcement strain and top compressive zone concrete strain versus fatigue life ratio n/N for the specimen beams loaded statically to the corresponding upper fatigue limit are shown in Figure 5b,c. It can be seen from the figures that the strains in the bottom tensile reinforcement and the concrete strains in the top compressive zone increase rapidly when the specimen beams are loaded to the corresponding fatigue upper limit within the first 10% of the cycles of fatigue life. Within 10–70% of the fatigue life, the strains develop steadily. After that, additional cyclic loading leads to rapid strain development.

The mid-span deflection versus fatigue life ratio n/N of the specimen beams when statically loaded to the corresponding upper fatigue limit is shown in Figure 5d. From Figure 5d, it can be seen that the change in the mid-span deflection of the specimen beams also shows a three-stage pattern. In the first stage, the mid-span deflection increases significantly. In the second stage, the mid-span deflection of the specimen beams changes relatively smoothly. After that, the mid-span deflection increases rapidly as the number of cycles continues to increase.

## 3. Method

### 3.1. Principle of the DBN

**Figure 6:**
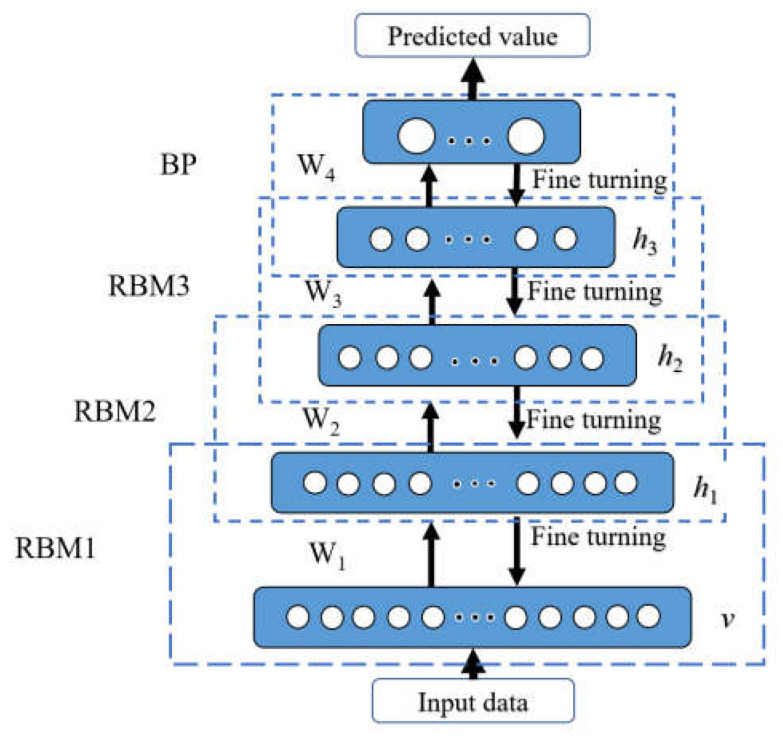
A prediction model based on the deep belief network (DBN).

The deep belief network is a neural network model consisting of multiple stacked restricted Boltzmann machines, as shown in Figure 6. The DBN training model mainly consists of two processes: pre-training and reverse fine-tuning. The pre-training process is a top-down independent and unsupervised learning process, where the output vector of the previous layer of the RBM network is used as the input vector of the next layer of the RBM network, and the deep feature information of the input vector data is extracted layer by layer to realize the training of the model layer by layer, to obtain the initialized network parameters. Then, the BP neural network is established in the last layer of DBN, and the output vector of the RBM network is used as the input vector of BP. The BP algorithm adjusts network parameters such as weights and biases, and to finish training the entire DBN, the error between the actual output and the desired output is propagated backward layer by layer.

A single RBM is made up of a visible layer and a hidden layer. Figure 6 depicts the layout of a network made up of a three-layer RBM, where *h* stands for the hidden layer, *v* for the visible layer, and W for the link weight between the two. Neurons in the same layer of the RBM are independent of one another, whereas connection weights connect neurons in adjacent layers. Binary values 0 and 1, respectively, reflect the inactive and active states of neurons.

The RBM is a thermodynamics-based energy model whose energy function can be expressed as:

(1) $$E(v,h|\theta )=-\sum_{i=1}^{n}\sum_{j=1}^{m}v_{i}w_{ij}h_{j}-\sum_{i=1}^{n}a_{i}v_{i}-\sum_{j=1}^{m}b_{j}h_{j}$$

where *v_i_* is the state of neuron *i* in the visible layer, *a_i_* is the offset corresponding to *v_i_*, *h_j_* is the state of neuron *j* in the hidden layer, *b_j_* is the offset corresponding to *h_j_*, *w_ij_* is the connection weight of neurons *i* and *j*, *θ* =(*w_ij_*,*a_i_*,*b_j_*) is the RBM parameter, *m* is the number of neurons in the hidden layer, and *n* is the number of neurons in the visible layer.

When the parameters in the RBM model are determined, the following joint probability distribution of (*v*,*h*) can be obtained:

(2) $$p(v,h|\theta )=\frac{1}{Z(\theta )}\exp(-E(v,h|\theta ))$$

where $Z(\theta )=\sum_{v}\sum_{h}\exp(-E(v,h|\theta ))$ is the normalization factor.

If the number of training samples is *N*, the parameter *θ* can be obtained by learning the maximum log-likelihood function of the samples:

(3) $$\theta^{*}=\underset{\theta }{\arg\max}L(\theta )=\underset{\theta }{\arg\max}\sum_{n=1}^{N}\log p(v^{n}|\theta )$$

where $p(v|\theta )=\frac{1}{Z(\theta )}\sum_{h}\exp(-E(v,h|\theta ))$ is the likelihood function of the data *v*.

The difficult normalizing factor *Z*(*θ*) computation is typically approximated using sampling techniques, such as Gibbs [40]. Hinton developed the contrastive divergence (CD) algorithm, which allows the *j*th neuron in the hidden layer to be determined from the state of the neuron in the visible layer and the *i*th neuron in the visible layer to be reconstructed from the hidden layer using the activation probabilities listed in Equations (4) and (5):

(4) $$p(h_{j}=1|v,\theta )=\sigma \left(b_{j}+\sum_{i}v_{i}w_{ij}\right)$$

(5) $$p(v_{i}=1|h,\theta )=\sigma \left(a_{i}+\sum_{j}w_{ij}h_{j}\right)$$

where $\sigma =\frac{1}{1+\exp(-x)}$ is the sigmoid activation function.

The maximum value of the log-likelihood function can be solved by the stochastic gradient ascent method. The amount of variation of parameters such as RBM weights and bias can be calculated as follows:

(6) $$\left\{\begin{array}{l} \Delta w_{ij}^{}=\varepsilon \left({\langle v_{i}h_{j}\rangle }_{data}-{\langle v_{i}h_{j}\rangle }_{reconstructed}\right)\\ \Delta a_{i}^{}=\varepsilon \left({\langle v_{i}\rangle }_{data}-{\langle v_{i}\rangle }_{reconstructed}\right)\\ \Delta b_{j}^{}=\varepsilon \left({\langle h_{j}\rangle }_{data}-{\langle h_{j}\rangle }_{reconstructed}\right) \end{array}\right.$$

where ${\langle •\rangle }_{data}$ is the distribution defined by the original data model,${\langle •\rangle }_{reconstructed}$ is the distribution defined by the reconstructed model, and ε is the learning rate.

### 3.2. Parameter Optimization based on the PSO Algorithm

The particle swarm optimization (PSO) originated from the study of the foraging behavior of bird flocks. The basic idea is to find the optimal solution through collaboration and information sharing among different individuals in a swarm. PSO treats each individual in a swarm as a particle without volume and mass in a multidimensional search space. The PSO algorithm first initializes a set of particles in the multidimensional search space and then iteratively updates its speed and direction according to its optimal value and the global optimal value of the population to output the optimal solution [41].

Set a population *X* = (*X*_1_, *X*_2_,···*X_n_*) consisting of *n* particles, and in the *D*-dimensional search space, each particle *n_i_* in this population has its velocity vector *V_i_* = (*V_i_*_1_, *V_i_*_2_,···*V_iD_*)^T^ and position vector *X_i_* = (*x_i_*_1_, *x_i_*_2_···*x_iD_*)^T^, and the particle’s superiority concerning the target is evaluated by its corresponding individual fitness value, which leads to the individual optimal solution *P_i_* = (*P_i_*_1_, *P_i_*_2_···*P_iD_*)^T^ when the particle flies in the *D*-dimensional search space. In turn, the individual optimal solution *P_i_* = (*P_i_*_1_, *P_i_*_2_···*P_iD_*)^T^ is obtained. The particle, when flying in the *D*-dimensional search space, will combine the flight experience of other particles and its previous flight state to adjust the next position and velocity, thus outputting the current global optimal solution *P_g_* = (*P_g1_*, *P_g_*···*P_gD_*)^T^ and obtaining the optimal solution by *k* iterations. The velocity and position of the particle are updated as follows:

(7) $$V_{id}^{k+1}=\omega V_{id}^{k}+c_{1}r_{1}\left(P_{id}^{k}-X_{id}^{k}\right)+c_{2}r_{2}\left(P_{gd}^{k}-X_{id}^{k}\right)$$

(8) $$X_{id}^{k+1}=X_{id}^{k}+V_{id}^{k+1}$$

where *c*_1_ and *c*_2_ are acceleration values, which are used to adjust the maximum step of individual and group optimal positions, *r*_1_ and *r*_2_ are inertia factors, distributed between [0,1], *ω* is the inertia weight, which can be used to balance the global search ability and local search ability, the velocity and position of particles are generally limited to the interval: $\left[-V_{\max},V_{\max}\right]$ and $\left[-X_{\max},X_{\max}\right]$, to avoid blindly searching for particles.

### 3.3. Fatigue Performance Prediction of RC Beams based on the PSO-DBN Model

**Figure 7:**
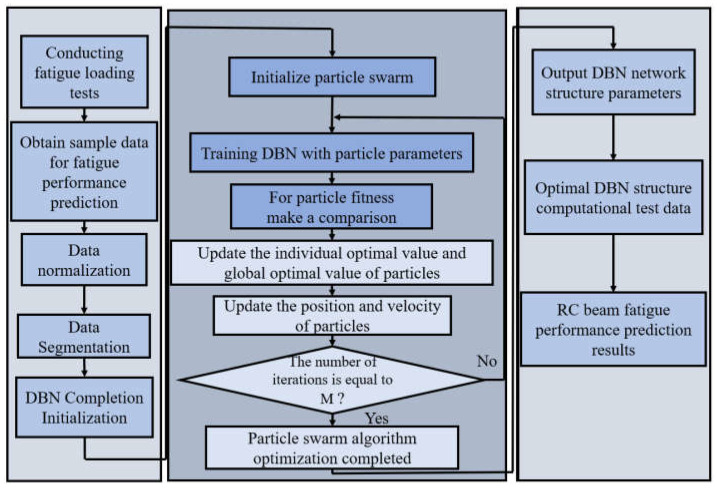
Fatigue performance prediction model of RC beams based on the PSO-DBN.

The flow chart of the fatigue performance prediction of RC beams based on the PSO-DBN model is shown in Figure 7. The specific steps of the prediction model are as follows:

In the first step, the data collected during the fatigue loading test are organized and pre-processed. The data are normalized by normalizing the original data to ensure that the data are relatively undistorted. After the normalization is completed, the data set is divided into two mutually exclusive sets, one of which is used as the training data set and the other as the test data set. After that, DBN initialization is completed.

In the second step, the initialization of particle position and velocity is completed, the particle fitness value is calculated, and then the particle position and velocity are updated. When the fitness value satisfies the set condition or the number of iterations is equal to M, the second step of PSO optimization is finished. Otherwise, the process of calculating the particle fitness value and updating the position and velocity of the particles will be repeated until the determination condition is satisfied.

In the third step, the DBN model structure parameters after PSO optimization are used to calculate the test data set by the optimal DBN structure and complete the prediction of the fatigue performance of the RC specimen beams.

### 3.4. Test Data Pre-Process

The data in this section were selected from the loading time range data of the RC specimen beams fatigue test. For the detailed data, see Table A1 in Appendix A. A total of 300 original data samples were selected as the input data for the PSO-DBN model based on 100-time course data for each of the different damage stages of the fatigue loading tests of RC-2, RC-4, and RC-6 specimen beams. In this study, based on the 300 experimental data samples, 70% of them were randomly selected as training sets, and the remaining 30% of data samples were used as test sets.

Based on the a priori knowledge of fatigue loading of RC beams, four relevant factors were identified as the input vectors of the model, namely: load amplitude (kN), concrete strength (MPa), static loading values at different damage stages (kN), and fatigue life ratio(n/N). Since the crack width time-range data were not available for the time being, the three mechanical property indices of concrete strain (*με*), tensile reinforcement strain (*με*), and mid-span deflection (mm) at the top of the RC specimen beams were used as the output vectors of the model in this paper.

Since the magnitudes of different variables were different, the data were normalized and mapped to the interval [0,1] before the formal experiments to prevent their variability from affecting the modeling effect, reduce the computational complexity, and accelerate the convergence speed. In this paper, the normalization method was used to linearly transform the original data, as in Equation (9):

(9) $$x_{i}^{*}=\frac{x_{i}-x_{\min}}{x_{\max}-x_{\min}}$$

where $x_{i}^{*}$ is the normalized value of the sample data, $x_{i}$ is the original value of the input variable, and $x_{\max}$ and $x_{\min}$ are the maximum and minimum values of the original data, respectively.

### 3.5. Evaluation Indicators

In this study, the model’s performance was assessed using the root mean square error (*RMSE*), mean absolute error (*MAE*), mean absolute percentage error (*MAPE*), and coefficient of determination (*R*^2^). The performance of the prediction increases with decreasing *RMSE*, *MAE*, and *MAPE*. *R*^2^ measures the percentage of the dependent variable’s total variation that the independent variable explains through the regression connection. It is generally accepted that the closer *R*^2^ is to 1, the better the regression relationship fits the data. The formula for calculating the above valuation metrics is shown below:

(10) $$RMSE=\sqrt{\frac{1}{N}\sum_{i=1}^{N}{\left(y_{i}-{\bar{y}}_{i}\right)}^{2}}$$

(11) $$MAE=\frac{1}{N}\sum_{i=1}^{N}\left|y_{i}-{\bar{y}}_{i}\right|$$

(12) $$MAPE=\frac{1}{N}\sum_{i=1}^{N}\left|\frac{y_{i}-{\bar{y}}_{i}}{y_{i}}\right|$$

(13) $$R^{2}=1-\frac{\sum_{i=1}^{N}{\left(y_{i}-{\bar{y}}_{i}\right)}^{2}}{\sum_{i=1}^{N}{\left(y_{i}-\bar{y}\right)}^{2}}$$

where $y_{i}$ is the actual sample value, ${\bar{y}}_{i}$ is the model predicted value, and *N* is the number of samples.

### 3.6. Model Parameter Setting

The selection of optimal parameters was achieved by using a longitudinal comparison method. Any three different test sample data sets were selected to compare the effect of the different number of hidden layers on model prediction. Each test sample set contains 90 data samples. The number of hidden layer nodes was set to 105, and the effect of different hidden layer choices on model prediction was analyzed, as shown in Table 3.

As can be seen from Table 1, when the number of hidden layers is less than four, the model error gradually decreases as the number of hidden layers increases, which indicates that the model prediction accuracy keeps improving. When the number of hidden layers is greater than four, the model error keeps increasing as the number of hidden layers increases, which is because too many hidden layers make the training of the model complicated and lead to the overfitting phenomenon. Therefore, the optimal number of hidden layers for this model is set to four.

The proper number of hidden layer nodes was discussed and decided upon when the number of hidden layers was established. The research discusses the implications of the prediction when there are 65–125 nodes, as indicated in Table 4. When there are fewer than 105 nodes, the model error gradually decreases as there are more nodes. When there are more than 105 nodes, the model error gradually rises with the number of nodes. Consequently, 105 nodes are needed for the best effect to be realized.

The parameters of the deep belief network model were automatically adjusted using the PSO algorithm, and the parameters of the model were output. The number of DBN hidden layer nodes was automatically output as (115, 129, 109, 105) after PSO completes the parameter optimization and converges. The number of particle swarm is 20, acceleration factor *c1* = *c2* = 1.49, learning rate is 0.01. The specific parameters of the model are taken as shown in Table 5.

## 4. Results and Discussion

### 4.1. Prediction Capability of the PSO–DBN Model

**Figure 8:**
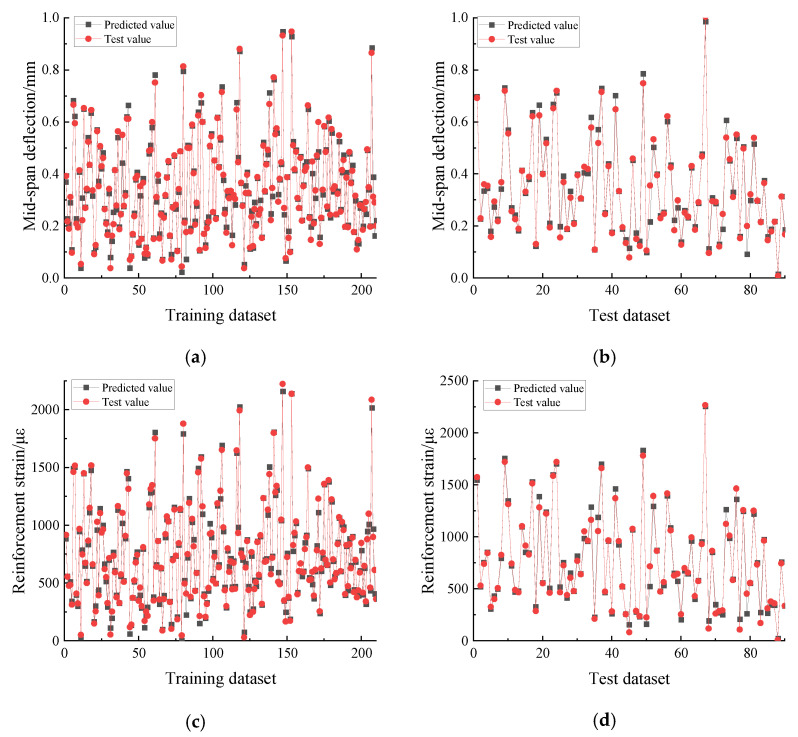

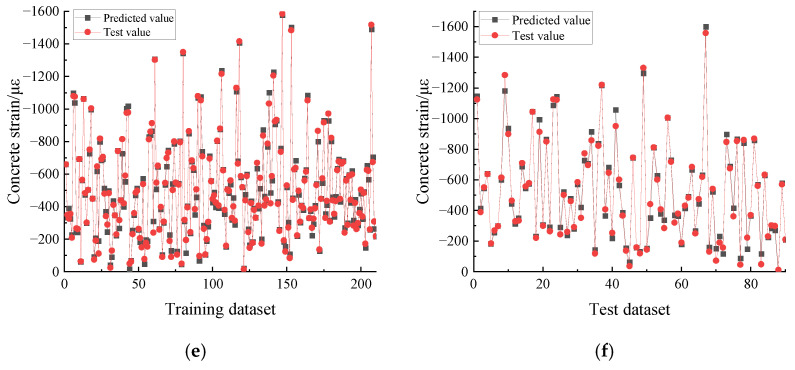
Comparison between predicted and experimental values of PSO-DBN model: (**a**) mid-span deflection prediction for training data; (**b**) mid-span deflection prediction for test data; (**c**) reinforcement strain prediction for training data; (**d**) reinforcement strain prediction for test data; (**e**) concrete strain prediction for training data; and (**f**) concrete strain prediction for test data.

In this section, the predicted results of the PSO-DBN model are evaluated. The comparison between the test values and the predicted values of the three output vectors (mid-span deflection, tensile reinforcement strain, and top compressive zone concrete strain) in the model is shown in Figure 8, including both the training and test data sets. The comparison shown in Figure 8 shows that the predicted values of mid-span deflection, tensile reinforcement strain, and concrete strain in the top compression zone of the RC specimens output from the model are in good agreement with the test values. The errors between the predicted and tested values for both the training and test datasets are relatively small, with the mean absolute percentage errors (*MAPE*) of 0.075, 0.111, and 0.124 for the test dataset part and 0.067, 0.075, and 0.071 for the training dataset part for the three output vectors, respectively. Error-values indicate that the PSO-DBN model proposed in this paper predicts well the mechanical properties of RC specimen beams under cyclic loading at different damage stages.

**Figure 9:**
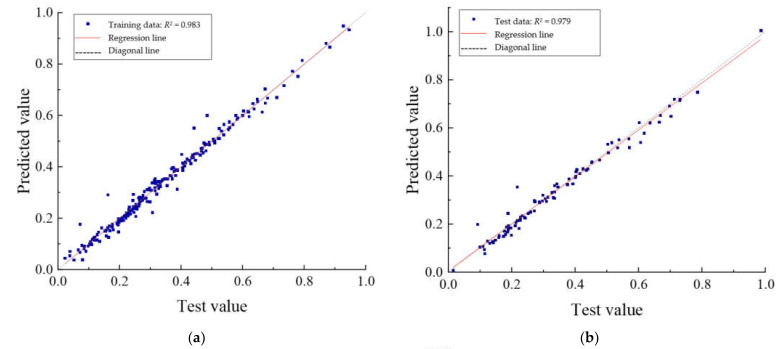

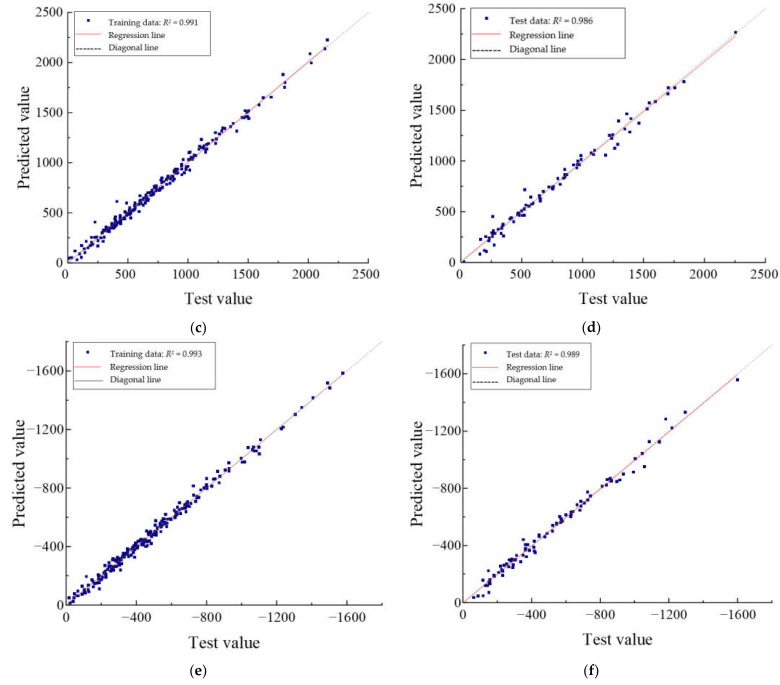
Correlation analysis between test and predicted values of fatigue performance of RC specimen beams: (**a**) mid-span deflection prediction for training data; (**b**) mid-span deflection prediction for test data; (**c**) reinforcement strain prediction for training data; (**d**) reinforcement strain prediction for test data; (**e**) concrete strain prediction for training data; and (**f**) concrete strain prediction for test data.

The regression plots of the two parts of the training set and the test set are shown in Figure 9. From Figure 9, it can be seen that the PSO-DBN model has a better prediction ability. For the training set, the coefficients of determination (*R*^2^) of the three output vectors are 0.983, 0.991, and 0.993, respectively. For the test set, the coefficients of determination (*R*^2^) of the three output vectors are 0.979, 0.986, and 0.989, respectively. The coefficients of determination of the three output vectors remain above 0.97 for both the training and test parts. Therefore, it is feasible to use the PSO-DBN model to predict the mechanical properties of RC beams under cyclic loading at different damage stages with high accuracy and low error. It can be applied to develop a numerical tool to evaluate the deterioration performance of RC structures.

**Figure 10:**
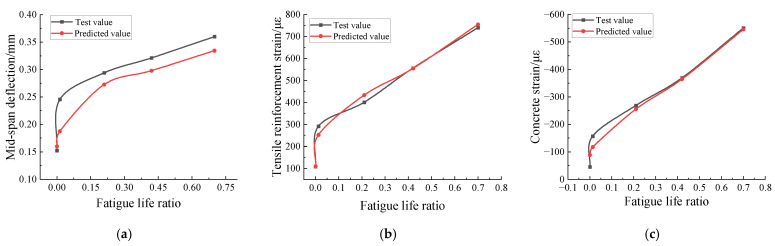
Analysis of fatigue loading process of RC-2 specimen beam: (**a**) mid-span deflection; (**b**) reinforcement strain; (**c**) concrete strain.

To illustrate the accuracy of the model in this paper in predicting the fatigue performance of RC beams, the model predicts the mid-span deflection, tensile reinforcement strain, and concrete strain in the compression zone of RC-2 specimen beam under different fatigue life ratios and compares them with the test values under the same load level (25 kN). As shown in Figure 10, the predicted values of the model are compared with the test values for a high degree of compliance. The predicted values develop rapidly at the fatigue life ratio of 0–0.15, and then the development rate slows down relatively around the fatigue life ratio of 0.15–0.7, which is consistent with the trend of the test results.

### 4.2. Models Comparison and Analysis

To highlight the efficiency of the PSO-DBN model, the prediction results of the PSO-DBN model, single DBN model, and BP model are compared.

**Figure 11:**
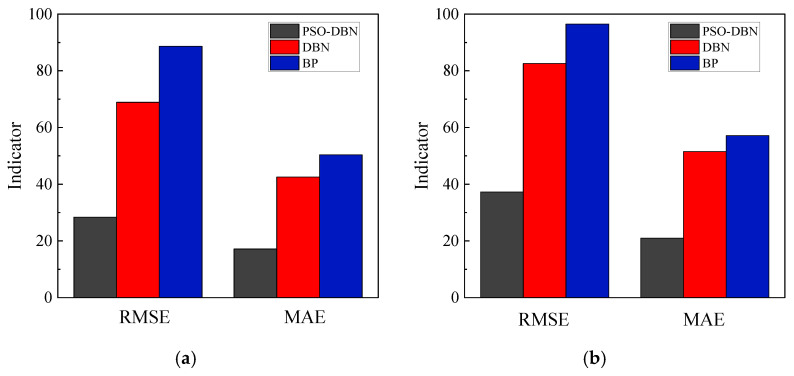

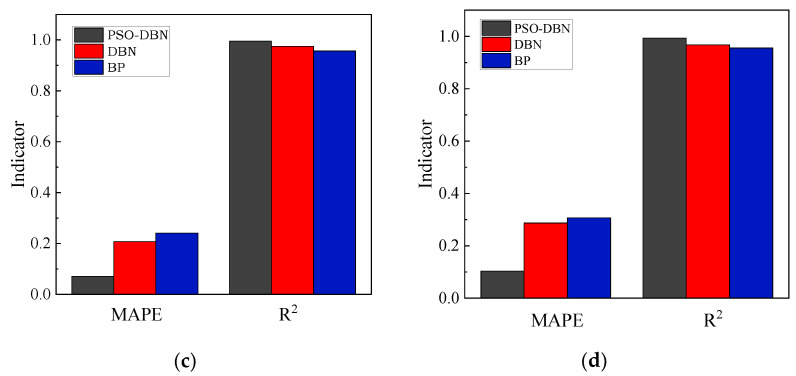
Models Comparison and Analysis:(**a**) *RMSE* and *MAE* for training data; (**b**) *RMSE* and *MAE* for testing data; (**c**) *MAPE* and *R*^2^ for training data; and (**d**) *MAPE* and *R*^2^ for testing data.

As shown in Figure 11, the correlation values of the two model algorithms in the training part (Figure 11a,c) and the testing part (Figure 11b,d) are determined considering *RMSE, MAE*, *MAPE*, and *R*^2^ as evaluation metrics. As can be seen in Figure 11, the PSO-DBN model is more accurate than the single *DBN* model and *BP* model, as evidenced by the reduction in the error values of *RMSE*, *MAE*, and *MAPE*, and the improvement in prediction accuracy is more pronounced in the training part than in the testing part. Considering the coefficient of determination (*R*^2^) as the fitted regression error criterion, the PSO-DBN model has *R*^2^ values closer to 1 compared with the DBN model without optimization and BP model and shows some superiority in both the training and test sets.

For comparison purposes, Table 6 and Table 7 show the exact values of the four error criteria when using the PSO-DBN and DBN models, respectively, where the numbers 1, 2, and 3 correspond to mid-span deflection, reinforcement strain, and concrete strain, respectively. Focusing on the test section, the average increase in *RMSE*, *MAE*, *MAPE*, and *R*^2^ reached 53.7%, 59.6%, 63.3%, and 6.0%, respectively. Thus, the use of PSO to adjust the weights and biases of DBN greatly improved the accuracy of the predictions.

## 5. Conclusion

In this paper, the fatigue performance of RC specimen beams was analyzed by conducting a four-point bending fatigue loading test under constant amplitude load, and the original database was established. A deep belief network (DBN) model for fatigue performance prediction of RC beams was established for the first time by using deep learning with massively parallel processing and self-learning characteristics. After training RBM layer by layer and extracting feature information from complex data, the fatigue performance prediction model of RC beams based on PSO-DBN was established by adaptively adjusting DBN model parameters using a particle swarm optimization (PSO) algorithm. The conclusions of this study are as follows.

1. Under the action of constantamplitude four-point bending cyclic loading, the mid-span deflection, tensile reinforcement strain, and concrete strain in the compression zone at the top of the beam of RC specimen beams with CRB600H for tensile reinforcement showed a three-stage trend at different damage stages with different static loading, i.e., rapid development at the initial stage, stable and slow development at the middle stage, and rapid development at the later stage until the fatigue fracture of the reinforcement.
2. The PSO-DBN model describes the complex nonlinear mapping relationship between the RC specimen beams and their material properties, load magnitude, and other factors and accurately predicts and reflects the real process of fatigue damage evolution of the RC specimen beams. By collecting the static loading time data of RC specimen beams at different damage stages during the fatigue loading test, a database containing 300 samples was established and used to train a DBN model. The parameters of the DBN model were adjusted by using PSO to establish the PSO-DBN model. Four evaluation metrics, namely root mean square error (*RMSE*), mean absolute error (*MAE*), mean absolute percentage error (*MAPE*), and coefficient of determination (*R*^2^), were used to evaluate the errors between the predicted values of the PSO-DBN model and test values. The prediction results of the PSO-DBN model showed high reliability, and in the three output vectors of the test section, the coefficient of determination (*R*^2^) reached 0.979, 0.986, and 0.989, respectively.
3. The prediction performance of the model on the development process of mid-span deflection, reinforcement strain, and concrete strain in the compressive zone under cyclic loading of the specimen beams was analyzed by using an RC-2 specimen beam as an example. The results showed that the predicted values of mid-span deflection, strain in the tensile reinforcement, and concrete strain in the compression zone of the specimen beam under static load (25 kN) at different fatigue life ratios do not differ significantly from the tested values, and the model prediction of the development trend is consistent with the test results.
4. The PSO-DBN model was compared with the single DBN model and BP model, and the comparison showed that the prediction performance of the PSO-DBN model is better, and the accuracy of *RMSE*, *MAE*, *MAPE*, and *R*^2^ are improved to different degrees. Focusing on the test set of the PSO-DBN model and the single DBN model, the average increases in *RMSE*, *MAE*, *MAPE*, and *R*^2^ reached 53.7%, 59.6%, 63.3%, and 6.0%, respectively. This indicated that the PSO-DBN model could predict the fatigue performance of RC specimen beams more efficiently and accurately.

## Figures and Tables

**Table 1:** Mixture ratio and type of concrete.

Design Strength	Cement (kg/m^3^)	Fly Ash (kg/m^3^)	Sand (kg/m^3^)	Rocks (kg/m^3^)	Water (kg/m^3^)	Additives (kg/m^3^)	Admixture (kg/m^3^)
C35	260	/	734	1101	160	7.8	112
C40	350	20	835	810	178	77.4	100
C60	365	65	713	1175	128	4.95	65

**Table 2:** Test design loading scheme.

Specimen Number	Design Strength	Load Range (kN)		Load Level *P_max_/P_u_*	Mode Failure
		*P_min_*	*P_max_*		
RC-1	C35	—	—	1.0	Static damage
RC-2	C35	10	40	0.20	Fatigue damage
RC-3	C40	—	—	1.0	Static damage
RC-4	C40	10	70	0.30	Fatigue damage
RC-5	C60	—	—	1.0	Static damage
RC-6	C60	10	90	0.27	Fatigue damage

**Table 3:** The effect of the choice of the number of hidden layers on the prediction effect of the model.

The Number of Hidden Layers	The Test Set 1-*MAPE*	The Test Set 2-*MAPE*	The Test Set 3-*MAPE*
2	0.128	0.123	0.122
3	0.126	0.122	0.118
4	0.116	0.115	0.114
5	0.118	0.116	0.116
6	0.122	0.120	0.121
7	0.123	0.123	0.123
8	0.129	0.128	0.126

**Table 4:** The effect of the number of nodes in the hidden layer on the prediction effect of the model.

The Number of Neurons	The Test Set 1-*MAPE*	The Test Set 2-*MAPE*	The Test Set 3-*MAPE*
65	0.125	0.126	0.124
100	0.123	0.119	0.118
105	0.115	0.117	0.113
110	0.118	0.121	0.116
115	0.123	0.122	0.118
125	0.128	0.123	0.124

**Table 5:** PSO-DBN model parameters.

Description	Symbol	Value
The number of neurons in the input layer	-	4
The number of neurons in the output layer	-	3
The number of RBMs	-	4
Iteration number of each RBM	-	100
The number of neurons in the first hidden layer	*h*_1_	115
The number of neurons in the second hidden layer	*h*_2_	129
The number of neurons in the third hidden layer	*h*_3_	109
The number of neurons in the fourth hidden layer	*h*_4_	105
The learning rate of the DBN	*η*	0.01
The momentum of the DBN	*α*	0.5
The acceleration factor of PSO	*c*_1_,*c*_2_	1.49
The iteration number of PSO	M	100
The inertia weight of PSO	*w*	0.9
The population factor of PSO	W	20

**Table 6:** Comparison of evaluation metrics between PSO-DBN model and single DBN model (a).

Data	Model	*RMSE*-1	*RMSE*-2	*RMSE*-3	*MAE*-1	*MAE*-2	*MAE*-3
Training	PSO-DBN	0.024	41.461	26.431	0.015	31.001	20.472
	DBN	0.059	94.186	73.280	0.045	70.766	56.783
	%Gain	+59.4	+56.0	+64.0	+65.7	+56.2	+60.6
Testing	PSO-DBN	0.027	54.416	34.776	0.018	36.956	25.922
	DBN	0.053	107.648	94.096	0.043	80.363	74.360
	%Gain	+48.6	+49.5	+63.0	+59.6	+54.0	+65.1

**Table 7:** Comparison of evaluation metrics between PSO-DBN model and single DBN model (b).

Data	Model	*MAPE*-1	*MAPE*-2	*MAPE*-3	*R*^2^-1	*R*^2^-2	*R*^2^-3
Training	PSO-DBN	0.067	0.075	0.071	0.983	0.991	0.993
	DBN	0.215	0.185	0.222	0.898	0.952	0.944
	%Gain	+68.9	+59.8	+68.2	+9.5	+4.1	+5.2
Testing	PSO-DBN	0.075	0.111	0.124	0.979	0.986	0.989
	DBN	0.221	0.250	0.390	0.921	0.946	0.921
	%Gain	+66.1	+55.6	+68.2	+6.3	+4.2	+7.4

## Data Availability

Not applicable.

## Appendix A

**Table A1:** The experimental data sets.

No.	Load Amplitude (kN)	Concrete Strength (MPa)	Static Loading Values (kN)	Fatigue Life Ratio(n/N)	Concrete Strain (με)	Tensile Reinforcement Strain (με)	Mid-Span Deflection (mm)
1	60	55	35	0.73	−660.207	917.4659	0.392024
2	80	63.7	15	0.3	−348.898	555.1427	0.217485
3	60	55	10	0.55	−325.581	475.9222	0.189237
4	30	36.9	25	0.35	−355.138	483.8292	0.311466
5	80	63.7	50	0	−208.525	313.328	0.096914
6	80	63.7	85	0.38	−1080.04	1461.95	0.666317
7	60	55	55	0.91	−1076.23	1516.539	0.594662
8	30	36.9	20	0.28	−266.424	408.7969	0.208433
9	60	55	25	0.0914	−262.274	323.9581	0.193996
10	60	55	45	0.64	−691.86	968.367	0.414213
11	60	55	20	0	−63.3075	52.00258	0.053224
12	60	55	25	0.46	−447.674	522.91	0.253336
13	30	36.9	35	0.42	−469.747	727.0375	0.451553
14	60	55	20	0.46	−425.065	495.1695	0.22686
15	30	36.9	40	0.7	−805.281	1190.168	0.61478
16	30	36.9	35	0.28	−406.872	636.4812	0.424396
17	80	63.7	65	0.53	−880.118	1298.979	0.539176
18	80	63.7	80	0.69	−1216.69	1653.203	0.715272
19	80	63.7	40	0.61	−622.718	982.8878	0.396408
20	60	55	35	0.1825	−379.845	438.0652	0.246948
21	30	36.9	20	0.07	−159.305	300.1294	0.173507
22	80	63.7	35	0.46	−503.693	801.0338	0.334273
23	60	55	40	0.365	−497.416	596.9282	0.306398
24	60	55	40	0.46	−560.724	687.382	0.334973
25	80	63.7	60	0	−330.364	447.4182	0.125569
26	80	63.7	40	0.23	−401.207	675.1316	0.318897
27	30	36.9	25	0.28	−313.223	450.194	0.303701
28	60	55	60	0.91	−1130.49	1648.27	0.647523
29	60	55	60	0.365	−664.729	933.771	0.445335
30	80	63.7	75	0.914	−1416.27	1994.367	0.880539
31	60	55	45	0.46	−587.855	755.7912	0.365869
32	80	63.7	80	0	−564.464	747.5251	0.221702
33	30	36.9	20	0.77	−520.255	724.4502	0.276353
34	30	36.9	10	0	−14.4425	31.04787	0.037254
35	60	55	50	0.1825	−438.63	634.1909	0.326397
36	80	63.7	55	0.23	−587.363	863.5034	0.396788
37	30	36.9	30	0.139	−243.594	439.8448	0.327171
38	30	36.9	0	0.42	−146.709	222.5097	0.116433
39	80	63.7	10	0.61	−421.279	768.7126	0.235399
40	30	36.9	0	0.49	−176.977	250.9702	0.122254
41	60	55	40	0.1825	−379.845	447.7179	0.264636
42	30	36.9	15	0.56	−331.381	509.7025	0.20048
43	80	63.7	80	0.46	−1060.82	1450.698	0.65326
44	60	55	45	0.55	−669.251	855.2599	0.38785
45	30	36.9	20	0.56	−406.139	571.7982	0.243361
46	80	63.7	0	0.83	−577.03	913.6663	0.263571
47	30	36.9	10	0.35	−172.779	320.8279	0.153683
48	60	55	55	0.73	−836.563	1236.105	0.508962
49	80	63.7	45	0.15	−406.738	684.7763	0.334508
50	30	36.9	35	0.35	−444.129	690.815	0.436039
51	80	63.7	60	0.46	−787.062	1134.657	0.492507
52	80	63.7	90	0.23	−1033.62	1442.919	0.669016
53	60	55	10	0.73	−420.543	575.3909	0.2222
54	30	36.9	20	0.7	−482.986	675.2911	0.268582
55	60	55	30	0.64	−587.855	717.8763	0.345753
56	80	63.7	65	0.914	−1205.56	1799.656	0.771727
57	80	63.7	70	0.46	−923.948	1286.304	0.552216
58	80	63.7	65	0.61	−933.446	1342.031	0.575364
59	60	55	45	0.1825	−416.021	511.5472	0.293324
60	30	36.9	35	0.07	−250.819	489.0039	0.381702
61	60	55	45	0.73	−737.08	1049.82	0.444983
62	80	63.7	90	0.83	−1586.06	2224.26	0.932645
63	30	36.9	25	0.07	−192.125	349.2885	0.268775
64	80	63.7	40	0	−123.614	168.0645	0.075977
65	30	36.9	30	0.21	−301.82	499.3532	0.342692
66	30	36.9	30	0.56	−530.029	724.4502	0.38732
67	60	55	10	0.365	−271.318	380.9899	0.154072
68	30	36.9	0	0.21	−83.833	173.3506	0.098968
69	80	63.7	80	0.914	−1484.71	2137.945	0.947851
70	30	36.9	40	0.28	−441.995	827.9431	0.50999
71	80	63.7	55	0.3	−629.749	935.2546	0.417464
72	60	55	65	0.2737	−637.597	1033.863	0.484995
73	30	36.9	30	0.07	−222.633	403.6223	0.307766
74	60	55	25	0.55	−497.416	604.2764	0.268725
75	30	36.9	40	0.07	−306.925	670.1164	0.461478
76	30	36.9	40	0.35	−502.542	866.7529	0.523577
77	30	36.9	15	0.7	−403.571	600.2587	0.219882
78	30	36.9	30	0.35	−392.646	592.4968	0.352401
79	60	55	60	0.1825	−560.724	793.6626	0.412368
80	60	55	60	0.2737	−614.987	897.6098	0.425548
81	80	63.7	75	0.61	−1053.91	1501.646	0.663472
82	80	63.7	20	0.23	−328.464	526.5123	0.230504
83	80	63.7	65	0	−382.39	530.4221	0.146402
84	30	36.9	40	0.013	−269.66	597.6714	0.447885
85	60	55	25	0.1825	−325.581	391.7876	0.209384
86	30	36.9	25	0.49	−390.063	615.7827	0.33668
87	80	63.7	40	0.76	−751.255	1150.314	0.43518
88	30	36.9	35	0.49	−532.615	783.9586	0.470952
89	80	63.7	90	0.015	−865.426	1230.836	0.599245
90	30	36.9	15	0.07	−135.765	256.1449	0.130619
91	30	36.9	15	0.77	−447.818	626.132	0.231531
92	60	55	35	0.55	−574.289	763.7191	0.339269
93	60	55	65	0.64	−917.959	1359.415	0.583906
94	30	36.9	40	0.139	−330.215	701.1643	0.484757
95	80	63.7	25	0.38	−435.185	658.9422	0.274556
96	60	55	70	0.55	−972.222	1391.721	0.616983
97	60	55	60	0	−307.494	504.2424	0.24312
98	60	55	70	0.64	−1003.88	1518.409	0.645558
99	60	55	70	0.365	−822.997	1224.365	0.57302
100	80	63.7	50	0.08	−438.25	705.5755	0.34238
101	30	36.9	30	0.28	−355.392	532.9884	0.352404
102	60	55	15	0.64	−434.109	585.0146	0.24433
103	60	55	15	0.91	−624.031	838.2156	0.323451
104	60	55	70	0.1825	−678.295	1070.56	0.548837
105	30	36.9	20	0.63	−448.065	602.8461	0.255001
106	80	63.7	45	0.61	−663.809	1030.794	0.414616
107	80	63.7	65	0.15	−673.632	959.3437	0.453897
108	60	55	55	0	−239.664	404.194	0.196857
109	30	36.9	25	0.56	−448.263	662.3545	0.336683
110	60	55	55	0.2737	−560.724	829.1861	0.385882
111	30	36.9	15	0.49	−287.127	473.4799	0.194658
112	30	36.9	35	0.56	−588.502	835.705	0.484542
113	30	36.9	30	0.42	−441.557	628.7193	0.364041
114	80	63.7	60	0.15	−598.368	893.8788	0.412404
115	30	36.9	10	0.63	−282.219	421.7335	0.176972
116	80	63.7	35	0.3	−423.016	700.5784	0.316189
117	80	63.7	55	0	−261.917	377.1777	0.109933
118	30	36.9	0	0.77	−298.063	437.2574	0.145537
119	80	63.7	30	0.23	−360.04	590.4656	0.279872
120	30	36.9	0	0.139	−74.5224	150.0647	0.091206
121	80	63.7	20	0.61	−504.851	847.0176	0.277024
122	60	55	15	0.365	−339.147	417.7019	0.184982
123	60	55	20	0.64	−483.85	648.9018	0.26422
124	30	36.9	30	0.013	−171.396	341.5265	0.292246
125	30	36.9	35	0.63	−625.755	879.6895	0.49425
126	80	63.7	0	0.914	−620.784	1100.229	0.348846
127	80	63.7	20	0.08	−260.09	461.1366	0.19692
128	80	63.7	90	0.76	−1517.7	2087.133	0.865453
129	80	63.7	90	0	−673.995	899.1677	0.312398
130	80	63.7	45	0.015	−308.294	613.0197	0.29058
131	30	36.9	0	0.56	-190.948	256.1449	0.126135
132	30	36.9	20	0.21	−217.519	362.2251	0.200671
133	80	63.7	85	0.46	−1125.17	1573.565	0.692161
134	60	55	15	0.55	−388.889	530.751	0.228942
135	30	36.9	25	0.7	−550.725	739.9741	0.359972
136	60	55	25	0.82	−637.597	843.9405	0.354446
137	30	36.9	15	0.21	−184.665	326.0026	0.157789
138	30	36.9	25	0.21	−268.976	401.0349	0.293999
139	80	63.7	20	0.15	−297.01	507.3762	0.217584
140	60	55	35	0.64	−614.987	827.0266	0.367848
141	60	55	65	0.91	−1284.24	1721.1	0.720159
142	30	36.9	40	0.49	−646.926	1029.754	0.564327
143	60	55	60	0.73	−899.871	1313.573	0.555243
144	80	63.7	20	0.53	−463.83	743.3757	0.256352
145	30	36.9	20	0.42	−329.307	489.0039	0.2259
146	30	36.9	10	0.7	−333.441	465.718	0.188612
147	80	63.7	30	0.83	−710.091	1102.311	0.41166
148	80	63.7	30	0.61	−556.948	915.7463	0.331556
149	80	63.7	50	0.3	−579.093	829.9471	0.388912
150	80	63.7	60	0.83	−1044.13	1514.141	0.621715
151	30	36.9	0	0.63	−221.22	287.1928	0.130013
152	80	63.7	90	0.08	−913.284	1283.451	0.625089
153	30	36.9	40	0	−111.316	393.273	0.352791
154	30	36.9	35	0.139	−299.727	553.6869	0.399166
155	60	55	60	0.64	−850.129	1223.162	0.517877
156	80	63.7	10	0.23	−264.031	462.5689	0.194059
157	60	55	65	0.82	−1125.97	1585.47	0.652035
158	80	63.7	60	0.914	−1123.44	1719.841	0.719911
159	80	63.7	0	0.3	−243.391	465.6075	0.155043
160	60	55	55	0.1825	−501.938	725.2099	0.368299
161	80	63.7	15	0.08	−260.019	440.3374	0.189057
162	60	55	45	0.2737	−461.24	606.4939	0.308701
163	80	63.7	15	0.15	−291.468	478.6043	0.207145
164	60	55	60	0.55	−818.475	1105.62	0.506877
165	30	36.9	30	0.63	−585.923	768.4347	0.395082
166	80	63.7	40	0.15	−351.977	640.0501	0.305977
167	60	55	35	0.82	−773.256	1053.11	0.427178
168	60	55	40	0.73	−696.382	963.2508	0.4185
169	80	63.7	85	0.08	−858.519	1163.771	0.578445
170	30	36.9	0	0.35	−118.762	212.1604	0.108668
171	80	63.7	80	0.08	−822.899	1055.271	0.518868
172	80	63.7	75	0.76	−1220.73	1661.099	0.715156
173	60	55	30	0.2737	−406.977	473.6757	0.244651
174	60	55	55	0.46	−646.641	964.874	0.429834
175	60	55	50	0.55	−691.86	937.2205	0.429704
176	60	55	20	0.0914	−253.23	282.6227	0.171915
177	80	63.7	90	0.15	−951.575	1371.154	0.648344
178	80	63.7	20	0.76	−601.937	960.232	0.333863
179	30	36.9	10	0.77	−366.052	522.6391	0.194437
180	30	36.9	10	0.21	−137.858	256.1449	0.134281
181	30	36.9	20	0	−35.8745	82.79431	0.07841
182	80	63.7	55	0.53	−745.969	1077.171	0.458812
183	30	36.9	15	0.139	−159.051	287.1928	0.150027
184	30	36.9	10	0.139	−119.225	238.0336	0.122632
185	80	63.7	75	0.83	−1331.49	1780.697	0.748748
186	80	63.7	70	0.08	−705.165	964.2066	0.461785
187	60	55	0	0.0914	−144.703	226.0837	0.105498
188	80	63.7	55	0.015	−441.049	716.811	0.355436
189	80	63.7	35	0.914	−812.722	1392.608	0.533239
190	60	55	50	0.46	−601.421	864.8691	0.394546
191	60	55	25	0.365	−406.977	473.1684	0.229157
192	80	63.7	35	0.08	−284.91	565.0405	0.246409
193	80	63.7	70	0.61	−1005.99	1417.061	0.621988
194	80	63.7	45	0.69	−718.497	1062.686	0.424956
195	80	63.7	0	0.53	−319.963	628.2452	0.183467
196	60	55	65	0	−379.845	644.9741	0.298164
197	60	55	15	0.73	−479.328	621.1758	0.26411
198	60	55	10	0.0914	−189.922	254.375	0.127698
199	80	63.7	20	0.46	−431.013	698.7271	0.251184
200	60	55	0	0.82	−483.85	655.6703	0.235172
201	80	63.7	55	0.38	−684.439	995.8504	0.43038
202	30	36.9	15	0.42	−249.858	429.4955	0.184953
203	60	55	35	0.365	−474.806	573.7242	0.290911
204	60	55	65	0.1825	−619.509	929.8723	0.467416
205	80	63.7	85	0.914	−1558.62	2267.204	1.004806
206	60	55	40	0	−131.137	117.5566	0.095419
207	80	63.7	20	0.69	−541.768	864.5592	0.295112
208	30	36.9	15	0.63	−340.703	551.0996	0.208239
209	30	36.9	35	0	−71.5098	261.3195	0.292439
210	60	55	0	0.2737	−189.922	284.8837	0.120883
211	30	36.9	25	0.013	−157.2	292.3674	0.245483
212	80	63.7	80	0.15	−847.513	1123.831	0.539548
213	80	63.7	60	0.3	−674.937	1011.873	0.456336
214	60	55	50	0.0914	−361.757	584.4493	0.311015
215	80	63.7	40	0.914	−853.809	1464.446	0.551451
216	30	36.9	25	0	−45.4206	108.6675	0.152333
217	80	63.7	50	0.76	−860.768	1257.274	0.497423
218	80	63.7	25	0.015	−221.873	453.2468	0.199628
219	60	55	15	0.1825	−289.406	327.2771	0.165199
220	30	36.9	25	0.42	−369.113	556.2743	0.321162
221	80	63.7	60	0.61	−869.106	1251.055	0.539027
222	60	55	15	0.82	−560.724	734.2249	0.297077
223	30	36.9	30	0	−47.9772	170.7633	0.214616
224	80	63.7	35	0.69	−633.598	963.6751	0.365276
225	60	55	10	0.1825	−230.62	313.175	0.145281
226	60	55	15	0.2737	−302.972	376.9897	0.178389
227	60	55	30	0.0914	−298.45	360.6411	0.216083
228	30	36.9	0	0	−11.6504	7.761966	0.007774
229	60	55	25	0.73	−569.767	744.4718	0.312684
230	30	36.9	25	0.63	−483.184	716.6882	0.342501
231	30	36.9	15	0.28	−210.283	336.3519	0.167488
232	30	36.9	15	0	−23.9923	54.33376	0.037457
233	30	36.9	20	0.013	−129.026	253.5576	0.161864
234	80	63.7	0	0.69	−410.211	735.0806	0.206719
235	30	36.9	35	0.21	−346.317	592.4968	0.414693
236	30	36.9	25	0.139	−229.379	377.749	0.278475
237	60	55	70	0.2737	−741.602	1165.551	0.564225
238	60	55	20	0.1825	−316.537	327.8714	0.187304
239	60	55	20	0.55	−438.63	576.5359	0.244439
240	80	63.7	85	0.015	−814.764	1107.967	0.550017
241	60	55	35	0.2737	−420.543	510.4022	0.27772
242	80	63.7	25	0.69	−591.067	909.2862	0.315891
243	60	55	65	0.73	−976.744	1449.898	0.612478
244	80	63.7	85	0.23	−978.863	1315.242	0.612036
245	30	36.9	0	0.013	−48.9041	119.0168	0.069858
246	30	36.9	0	0.07	−62.8757	139.7154	0.081504
247	30	36.9	10	0.49	−230.99	372.5744	0.169207
248	30	36.9	20	0.49	−359.571	522.6391	0.23754
249	30	36.9	30	0.49	−485.801	677.8784	0.383442
250	60	55	60	0.0914	−497.416	743.921	0.392581
251	30	36.9	0	0.7	−263.138	346.7012	0.135834
252	30	36.9	35	0.013	−206.59	460.5433	0.352595
253	30	36.9	10	0.28	−149.493	300.1294	0.149805
254	60	55	50	0.365	−538.114	810.6055	0.365968
255	30	36.9	10	0.013	−77.3182	173.3506	0.093525
256	60	55	0	0.1825	−176.357	253.2155	0.114289
257	80	63.7	45	0	−153.762	215.9824	0.091605
258	60	55	45	0.82	−813.953	1180.914	0.488939
259	80	63.7	45	0.83	−860.708	1314.618	0.49214
260	60	55	70	0.46	−913.437	1346.487	0.5994
261	60	55	0	0.46	−239.664	348.1767	0.149454
262	80	63.7	80	0.76	−1302.84	1752.072	0.75146
263	60	55	30	0.55	−547.158	641.0174	0.310592
264	60	55	40	0.64	−651.163	863.7965	0.396525
265	60	55	10	0.2737	−257.752	358.3801	0.151878
266	80	63.7	20	0.38	−398.196	631.7532	0.243432
267	60	55	30	0	−90.4393	89.29429	0.066604
268	60	55	35	0.0914	−307.494	361.2064	0.231552
269	80	63.7	15	0.76	−545.795	923.4785	0.318252
270	80	63.7	25	0.83	−699.089	1076.716	0.385663
271	60	55	50	0.64	−732.558	1032.153	0.449484
272	30	36.9	10	0.42	−189.083	336.3519	0.163386
273	60	55	35	0	−90.4393	103.4109	0.071123
274	60	55	10	0.82	−501.938	697.4839	0.270548
275	80	63.7	50	0.69	−800.603	1136.097	0.471592
276	60	55	0	0.91	−547.158	737.0656	0.281322
277	30	36.9	0	0.28	−104.79	186.2872	0.102846
278	80	63.7	35	0.53	−535.146	845.6807	0.342024
279	60	55	55	0.64	−795.866	1141.158	0.486981
280	60	55	15	0	−49.7416	46.91537	0.044319
281	80	63.7	90	0.61	−1350.86	1879.832	0.813765
282	80	63.7	15	0.23	−318.818	496.146	0.212317
283	80	63.7	15	0.015	−194.387	408.445	0.176141
284	30	36.9	40	0.21	−395.415	750.3234	0.500284
285	80	63.7	55	0.69	−863.565	1193.574	0.50016
286	30	36.9	15	0.35	−242.891	380.3364	0.179131
287	30	36.9	40	0.56	−684.183	1055.627	0.589556
288	30	36.9	30	0.7	−637.152	817.5938	0.402844
289	60	55	20	0.365	−384.367	431.8475	0.204882
290	60	55	30	0.46	−506.46	586.7393	0.288611
291	80	63.7	70	0.69	−1079.83	1458.513	0.624568
292	30	36.9	15	0.013	−96.1786	214.7477	0.107333
293	60	55	70	0.73	−1053.62	1577.165	0.702711
294	30	36.9	40	0.63	−709.805	1164.295	0.599259
295	30	36.9	10	0.56	−263.59	385.511	0.169207
296	30	36.9	10	0.07	−105.254	206.9858	0.11487
297	30	36.9	20	0.139	−198.898	318.2406	0.190971
298	30	36.9	20	0.35	−306.021	457.956	0.216198
299	30	36.9	35	0.7	−695.62	926.2613	0.507831
300	30	36.9	40	0.42	−556.114	934.0233	0.544925
